# Chromatographic Profile and Redox-Modulating Capacity of Methanol Extract from Seeds of *Ginkgo biloba* L. Originating from Plovdiv Region in Bulgaria

**DOI:** 10.3390/life12060878

**Published:** 2022-06-12

**Authors:** Lubomir Petrov, Albena Alexandrova, Mariana Argirova, Teodora Tomova, Almira Georgieva, Elina Tsvetanova, Milka Mileva

**Affiliations:** 1 National Sports Academy “Vassil Levski”, 21, Acad. Stefan Mladenov, Studentski Grad, 1700 Sofia, Bulgaria; dr.lubomir.petrov@gmail.com; 2 Department of Chemical Sciences, Faculty of Pharmacy, Medical University of Plovdiv, 4000 Plovdiv, Bulgaria; mariyana.argirova@mu-plovdiv.bg (M.A.); teodora.tomova@mu-plovdiv.bg (T.T.); 3 Institute of Neurobiology, Bulgaria Academy of Sciences, 23 Acad. G. Bonchev Str., 1113 Sofia, Bulgaria; al.georgieva@inb.bas.bg (A.G.); elinaroum@yahoo.com (E.T.); 4 The Stephan Angeloff Institute of Microbiology, Bulgarian Academy of Sciences, 26 Acad. G. Bontchev Str., 1113 Sofia, Bulgaria

**Keywords:** *Ginkgo biloba* seeds extract, phytochemical profile, antiradical activity, metal-reducing and metal chelating effect

## Abstract

Oxidative stress underlies the pathogenesis of many diseases, which determines the interest in natural substances with antioxidant properties. *Ginkgo biloba* L. leaves are well known and widely used in the pharmaceutical industry, but the therapeutic properties of the seeds are less studied. This study aimed to identify the chromatographic profile and to evaluate the antioxidant properties of methanol extract from seeds of *G. biloba* (GBSE). In the GBSE, flavonoids and terpenes were found as terpenes predominated. The GBSE antioxidant capacity determined by 2,2 azino-bis (3-ethyl-benzothiazoline-6-sulfonic acid) (ABTS) and 1-diphenyl-2-picrylhydrazyl (DPPH) methods were equal to 1.34% and 0.58% of the activity of reference substance Trolox, respectively. The results of the ferric reducing antioxidant power method showed that the effect of concentration 1 mg/mL (*w*/*v*) GBSE was equal to 7.418 mM FeSO_4_ used as a standard. The cupric reducing antioxidant capacity activity of the GBSE was found to be 215.39 µmol Trolox/g GBSE and is presented as Trolox equivalent. The metal chelation effect of 1 mg/mL (*w*/*v*) GBSE was equal to that obtained for 0.018 mM EDTA. In conclusion, GBSE showed a good ability to neutralize ABTS and DPPH radicals and could have a beneficial effect in pathological conditions with oxidative stress etiology.

## 1. Introduction

*Ginkgo biloba* L. is one of the oldest tree species on Earth, having existed for more than 200 million years. The beneficial properties of extracts from the leaves and seeds of the plant have been known and used to treat and maintain the well-being of people since ancient times. *G. biloba* tree is widely cultivated around the world. In Bulgaria, it has been cultivated since the end of the 19th century, and Plovdiv is the city with the most specimens [1,2].

Although the leaves, seeds, and nuts are often used in traditional Chinese medicine, modern research primarily focuses on ginkgo leaf extracts [3]. One of the reasons for avoiding seeds is the unpleasant odor emitted by ripened fleshy seed coats. Nevertheless, the seeds are rich in secondary metabolites, which are considered responsible for the beneficial properties of ginkgo leaves [4]. The study of the pharmacological properties of seed extracts may offer new perspectives for the use of these neglected sources of biologically active substances.

*Ginkgo biloba* leaf extracts have been found to improve mainly attention/concentration and memory, and they are part of several medications and supplements with neuroprotective effects [5,6]. Along with the possibility of using extracts for the treatment of neurodegenerative disorders such as dementia [7], Alzheimer’s disease [7,8], and Parkinson’s disease [9], pronounced positive effects on anxiety, schizophrenia, and depression have been found [10]. Extracts from the leaves of the plant improve cardiovascular and peripheral vascular disorders by improving blood circulation, strengthening capillary walls, and reducing clot formation [11,12]. Modern pharmacological studies have revealed that *G. biloba* extracts and individual substances extracted from the plant have an antitumor effect [13]. In addition, they are used against asthma and bronchitis [14] and have an anti-inflammatory effect [15].

The broad spectrum of compounds is responsible for the therapeutic effects of *G. biloba* extracts [16]. Among them, flavonol glycosides and terpene trilactones are present in high concentrations [17]. The significant levels of flavonoids and terpenoids determine the manifestation of antioxidant and radical-scavenger properties of extracts. These activities are based on their ability to be excellent donors of hydrogen, which is accepted by reactive radicals to yield much less active radical and non-radical species [18]. The interest in various substances with antioxidant properties stems from the participation of oxidative stress (OS) in many pathologies. Oxidative stress is a condition characterized by an increase in pro-oxidant processes and/or a decrease in antioxidant protection, leading to a pro/antioxidant imbalance in cells with consequences at higher organism levels. In living cells under normal physiological conditions, there is a balance between the rate of generation of reactive oxygen species (ROS) and their neutralization by the cell’s antioxidants [19,20]. The redox state of a cell determines its cellular functioning and is usually kept within a narrow range under normal conditions [19]. The fine balance between the useful and detrimental effects of ROS is due to the metabolic reactions that use oxygen and constitute a very important part of protecting living organisms and maintaining “redox homeostasis” by controlling the redox regulation in vivo [21]. In principle, the antioxidant effects of complex plant extracts can include various mechanisms of antioxidant defense such as free radical scavenging, the termination of oxidative chain reactions, reducing capacity, and binding of pro-oxidant metal ions [22]. Numerous studies have shown that a wide range of diseases such as cardiovascular, neurodegenerative, metabolic, pulmonary, renal, and neoplasms have OS etiology [23] and the application of antioxidants from plant or animal origin has beneficial effects; therefore, the search for new sources of antioxidants with therapeutic potential is important for human health.

The review of the existing data shows that most studies have focused on the effects of *G. biloba* leaf extracts, while the pharmacological effects of seed extracts have been poorly studied. In this context, the aim of this study was to investigate the phytochemical profile and the redox modulating potential—radical-scavenging, metal-reducing, and metal-chelating properties of methanol extract from seeds of *G. biloba* growing in Bulgaria’s Plovdiv region.

## 2. Materials and Methods

### 2.1. Preparation of Ginkgo biloba Seed Extract (GBSE)

Mature seeds of *Ginkgo biloba*, from trees grown on the campus of the Medical University in Plovdiv, Bulgaria. The seeds were peeled, and the endosperm was homogenized in a high-speed tissue homogenizer. The resulting slurry was mixed with anhydrous methanol (1:10 *w*/*v*) and stirred for 12 h at room temperature in a light-protected flask. The extract was centrifuged at 6000× *g* for 10 min, and the supernatant was separated. The extraction was carried out two more times, using 70% methanol as an extractant [4]. Thereafter, the combined extracts were evaporated at 30 °C under a vacuum [4]. The amount of dry matter obtained was determined gravimetrically. For the purposes of the present study, the dry extract was dissolved in distilled water. From the obtained *Ginkgo biloba* seed extract (GBSE) serial dilutions: 2.0, 1.0, 0.5, 0.25, 0.125, and 0.0625 mg/mL were prepared.

### 2.2. Chromatographic Determination of GBSE Composition

The GBSE composition was determined as 12 batches with an average dry matter content of 6–8% and was quantified by liquid chromatography-mass spectrometry (LC-MS) as described by Tomova et al. [4]. The chromatographic system Thermo Dionex Ultimate 3000 with a triple quadrupole mass detector, Thermo TSQ Quantum Access MAX and HESI-ionizator (Heated Electrospray Ionization) was used. The analytical conditions are given in Table 1.

### 2.3. ABTS^•+^ Assay

ABTS (2,2’-azino-bis (3-ethylbenzthiazoline-6-sulphonic acid) (7.0 mM) was mixed with potassium persulfate (2.45 mM) to obtain the cationic radical (ABTS^•+^) [24]. The solution was diluted in methanol (2 mL ABTS^•+^ + 58 mL methanol). The absorption at 743 nm of the resulting working solution was 1.1 ± 0.02 AU. Then, 1.425 mL of ABTS^•+^ working solution was added to 75 μL of the test extract, and the absorption at 743 nm was measured after 15 min of incubation at 37 °C against methanol. A blank containing 75 μL of water instead of the test extract also was measured against methanol. A calibration curve was obtained with different concentrations (10; 20; 30; 40; 50 mM) of Trolox dissolved in methanol. The antioxidant activity of the test substance relative to that of Trolox was calculated as the ratio between the concentrations of the test substance and of the Trolox, which gave 50% inhibition of the cationic radical ABTS^•+^.

### 2.4. DPPH Assay

DPPH analysis was conducted by the method described by Brand-Williams et al. [25]. First, 500 μL of the test solutions were added to 500 μL of a freshly prepared solution of 0.1 mM DPPH in methanol and incubated in the dark for 30 min. Then, the absorbance at 517 nm (A_517_) was measured against a mixture of DPPH solution and methanol (1:1) as a control. Antioxidant activity determined by the DPPH was calculated as follows:

Antioxidant activity (%) = [(A_517_ control − A_517_ probe)/A_517_ control] × 100

A calibration curve with different concentrations (10; 20; 30; 40; 50 mM) of Trolox dissolved in methanol was also obtained. The antioxidant activity of the test substance relative to that of Trolox was calculated as the ratio between the concentrations of the test substance and Trolox that gave 50% inhibition of the DPPH radical.

### 2.5. FRAP Assay

FRAP analysis was performed according to Benzie et al. [26] with some modifications. The following solutions: 0.03 M acetate buffer, 1.0 mM TPTZ, and 1.5 mM FeCl_3_ were mixed in the ratio 10:1:20, respectively. To 1.5 mL of the reaction mixture, 50 µL of the sample was added. After incubation for 4 min at 37 °C, absorbance was measured at 593 nm against the blank sample. A calibration curve was prepared with FeSO_4_, instead of a sample in concentrations: 0.1, 0.2, 0.3, 0.4, 0.5, 0.6, 0.7, 0.8, 0.9, and 1.0 mM. The antioxidant activity of GBSE was expressed as FRAP [mM Fe^2+^/1 g GBSE].

### 2.6. CUPRAC Assay

The CUPRAC analysis was performed according to Apak et al. [27] with modifications. The following solutions 10 mM CuCl_2_ in ddH_2_O, 1.0 M ammonium acetate buffer; pH 7.0, and 7.5 mM neocuproin (NC) in 96% ethanol were mixed in the ratio of 1:1:1. The tested substances were pipetted to Eppendorf tubes, and the volume was adjusted to 0.550 mL by adding H_2_O. Then, 1.5 mL of the reaction mixture was added. After incubation at 50 °C for 20 min, absorbance was measured at 450 nm against blank (1.5 mL reaction mixture and 0.550 mL H_2_O). The standard curve was prepared with Trolox at various concentrations ranging from 0.1 to 1.0 mM, and the results were expressed as CUPRAC (µM Trolox)/g GBSE).

### 2.7. Metal Chelating Assay

Metal chelation assay was applied after Venditti et al. [28]. A total of 0.2 mL of the sample solution, containing different concentrations of GBSE and reaction mixture, containing 0.74 mL of 0.1 M acetate buffer (pH 5.25), and 0.02 mL of 2 mM ferrous sulfate solution in 0.2 M hydrochloric acid were mixed for 10–15 s. Then, 0.04 mL ferrozine solution (5 mM) was added, and after incubation in the dark for 10 min at room temperature, the absorbance of the mixture at 562 nm was measured against the appropriate blank. The metal chelating capacity was determined using the following formula: Activity (%) = 100 (Ac − As)/(Ac), where Ac is the absorbance of the control and As is the absorbance of the sample solution. EDTA was used as a positive control, and the metal chelation capacity of GBSE was expressed as (mmol EDTA)/(g GBSE).

### 2.8. Statistics

All measurements were made in triplicate and the data in graphics were presented as mean ± standard deviation.

## 3. Results

### 3.1. Chromatographic Determination of GBSE Composition

**Figure 1:**
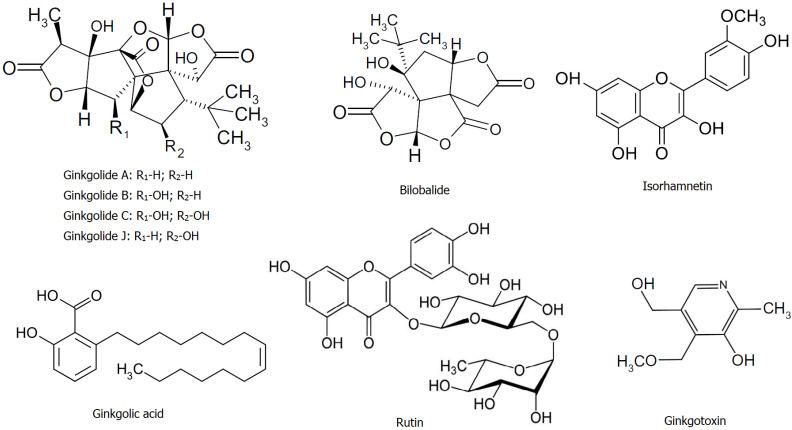
The main bioactive compounds in GBSE.

The chromatographic analysis by LC-MS showed the presence of flavonoids (quercetin, isorhamnetin, and rutin) and terpenes (ginkgolides A, B, C, J, and bilobalide), as well as ginkgotoxin (4′-O-methylpyrodoxin), and ginkolic acid (Figure 1). The average levels of these bioactive constituents of the extracts are shown in Table 2.

From the group of flavonoids, three representatives were identified, the largest amount being the glycoside flavonol rutin (quercetin-3-O-rutinoside) and a trace amount of its aglycone quercetin. The flavonol glycoside isorhamnetin was also detected (see Table 2 and chromatogram in Supplementary Material Figure S1).

Ginkgo-specific terpenes have been found to contain the largest amounts. The isomeric ginkgolides B and J have the highest content (221.44 μg/g), followed by ginkgolide A and bilobalide in almost equal concentrations (see Table 2 and chromatograms in Supplementary Material, Figure S2).

The toxic phenolic compounds, ginkgolic acid, and ginkgotoxin were also present in the studied GBSE see Table 2 and chromatograms in Supplementary Material, Figures S3 and S4). Ginkgolic acid isomers are lipophilic compounds, and after extraction with hexane, this amount was reduced to below 4 µg/g without significant changes in the levels of flavonoids and terpene trilactones. This extract was used thereafter in the analyses of the redox-modulating capacity of the studied GBSE.

### 3.2. ABTS-Radical-Scavenging Properties

**Figure 2:**
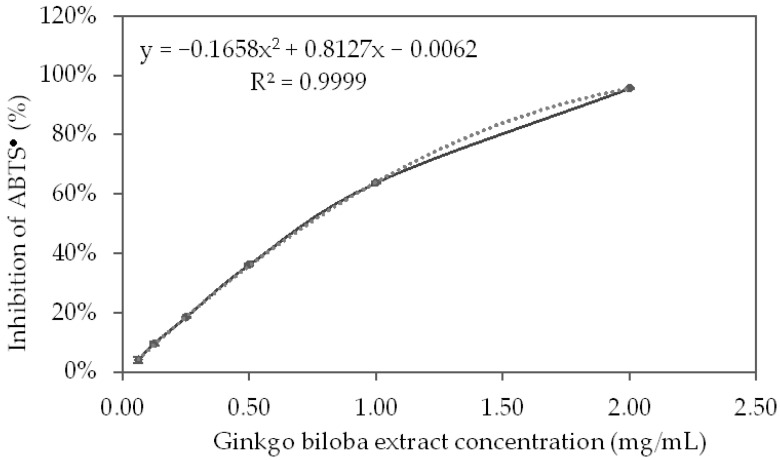
ABTS^•+^ inhibition curve from different concentrations of the *Ginkgo biloba* seed extract.

The results obtained for the inhibition of ABTS radicals (ABTS^•+^) by the tested GBSE concentrations are presented in Figure 2. As can see in the graphic, the antiradical activity of the extract showed a strict dose–response relationship in the concentration range of 0.5–2.5 mg/mL.

Based on the polynomial, which described the curve of inhibition of the ABTS^•+^ by GBSE, the IC_50_ of the GBSE was calculated:

GBSE IC_50_ = 728.2 mg/L

Using the linear equation of the calibration curve of inhibition of the ABTS^•+^ by Trolox (R^2^ = 0.9998) the concentration of Trolox (MW = 250.9), which leads to 50% inhibition of the ABTS^•+^, was calculated:

Trolox IC_50_ = 39.020 μM = 9.7663 mg/L

Trolox equivalent antioxidant capacity (TEAC%) of GBSE was computed as follow:

TEAC% = 100 × (Trolox IC_50_)/(GBSE IC_50_)

GBSE (TEAC%) = 100 × 9.7663/728.2 = 1.34%

This experiment showed that the antioxidant capacity of GBSE determined by the ABTS method was equal to 1.34% of that of the same amount of Trolox.

### 3.3. DPPH-Radical-Scavenging Properties

**Figure 3:**
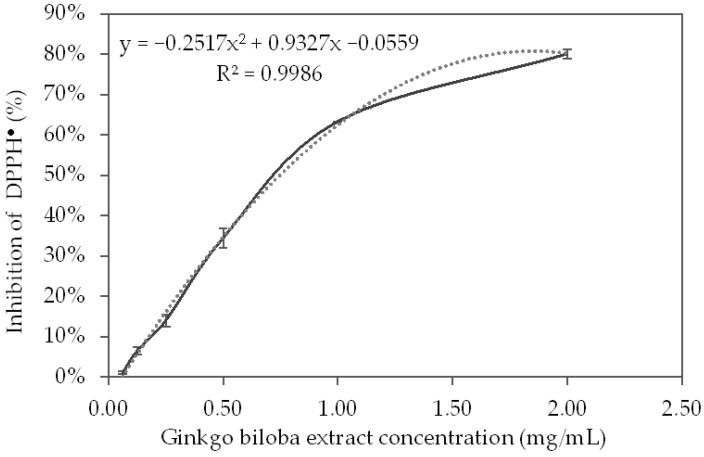
DPPH^●^ inhibition curve from different concentrations of the *Ginkgo biloba* seed extract.

The results of the DPPH radicals (DPPH^●^) inhibition by the different GBSE concentrations are presented in Figure 3. In the concentration range of 0–1.0 mg/mL, the radical-cleansing effect of GBSE increased to almost 70%, and the trend of the effect followed that of the reference substance Trolox. After this concentration, the effect of the extract was slightly lower than that of the standard.

Based on the polynomial, which described the inhibition curve of the DPPH^●^ (R^2^ = 0.9986), the concentration of GBSE, which leads to 50% inhibition of the DPPH^●^, was calculated:

GBSE IC_50_ = 746 μg/mL = 746 mg/L

Based on the linear equation of the calibration curve of DPPH^●^ inhibition by Trolox (R^2^ = 0.9997), we calculated the concentration of Trolox (MW = 250.9), which leads to 50% inhibition of the DPPH^●^:

Trolox IC_50_ = 17.28 μM = 4.32 mg/L

Trolox equivalent antioxidant capacity (TEAC%) of GBSE was calculated as follows:

TEAC% = 100 × (IC_50_ on Trolox)/(IC_50_ on GBSE)

GBSE (TEAC%) = 100 × 4.32/746.0 = 0.58%

Thus, the experiment showed that the antioxidant capacity of GBSE determined by the DPPH method was equal to 0.58% of that of the same amount of Trolox.

### 3.4. FRAP Assay

**Figure 4:**
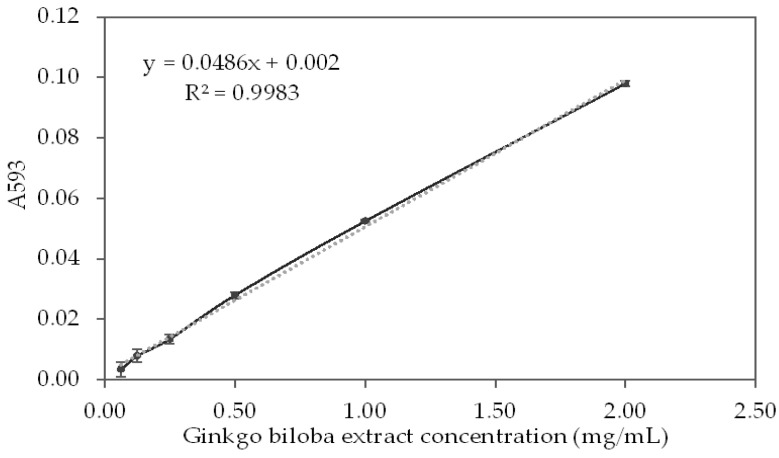
Reduction curve of Fe^3+^ in complex with TPTZ from different concentrations of the *Ginkgo biloba* seed extract.

The results for the degree of reduction of Fe^3+^ in complex with TPTZ to Fe^2+^ from GBSE are presented in Figure 4.

The reduction curve of Fe^3+^ to Fe^2+^ in a complex with TPTZ (described by A_593_) demonstrates a well-defined concentration dependence on the effect in the studied concentration range. The relation of the different concentrations of GBSE is described (R^2^ = 0.9983) with the following linear correlation equation.

A_593_ = 0.0486x − 0.0002; where x is GBSE concentration (mg/mL) (1)

The calibration curve of the FRAP method with different concentrations of FeSO_4_ is described with the following linear correlation equation:

A_593_ = 6.8883x − 0.0021; where x is FeSO_4_ concentration (mmol/L) (R^2^ = 0.9967)

From Equation (1), we calculated that the absorption of concentration of GBSE = 1.0 mg/mL is A_593_ = 0.0484, and by substituting A_593_ with this value in the above equation, we found that the effect of 1 mg/mL GBSE was equal to FeSO_4_ having concentration of 7.33 mM.

### 3.5. Cupric Reducing Antioxidant Capacity—CUPRAC

**Figure 5:**
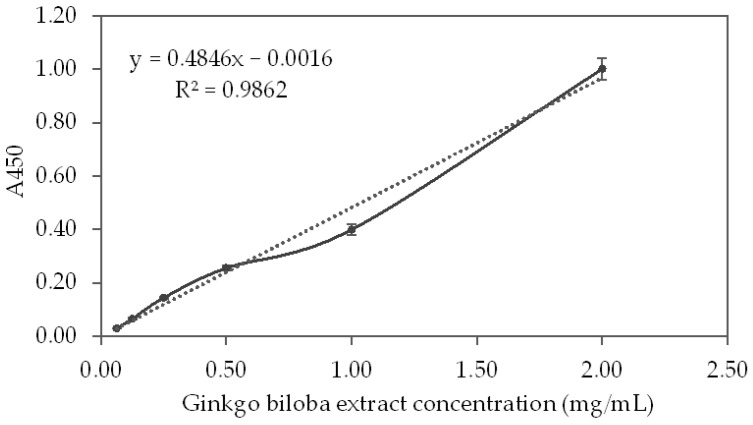
Neocuproine-Cu^+^ complex concentration resulting from Cu^2+^ reduction by different concentrations of the *Ginkgo biloba* seed extract.

The CUPRAC method is based on the absorbance measurement of Cu^1+^-neocuproine (Nc) chelate formed as a result of the redox reaction of chain-breaking antioxidants with the CUPRAC reagent, Cu^2+^-Nc, where absorbance is recorded at the maximal light-absorption wavelength of 450 nm [29]. The results for the degree of reduction of Cu^2+^ by GBSE, measured by chelation of the resulting Cu^+^ with neocuproine are presented in Figure 5.

Antioxidant index for GBSE using its Cu^2+^/Cu^+^ reducing capability measured in the presence of neocuproine is described with the following linear equation (R^2^ = 0.9862):

A_450_ = 0.04846x − 0.0016; where x is GBSE concentration (mg/mL) (2)

The calibration line obtained of the CUPRAC method with different concentrations of Trolox is described with the following linear correlation equation:

A_450_ = 0.0023x − 0.0124; where x is Trolox concentration (µmol/L) (R^2^ = 0.9981)

From Equation (2) it was found that at a concentration of GBSE = 1.0 mg/mL, the calculated absorption was A_450_ = 0.483. By substituting A_450_ in the above equation with the resulting value, we obtained that the effect of 1 mg/mL GBSE was equal to that which would be obtained at a concentration of Trolox = 215.39 µM, or CUPRAC = 215.39 (µmol Trolox)/(g GBSE).

### 3.6. Metal Chelation Capacity

**Figure 6:**
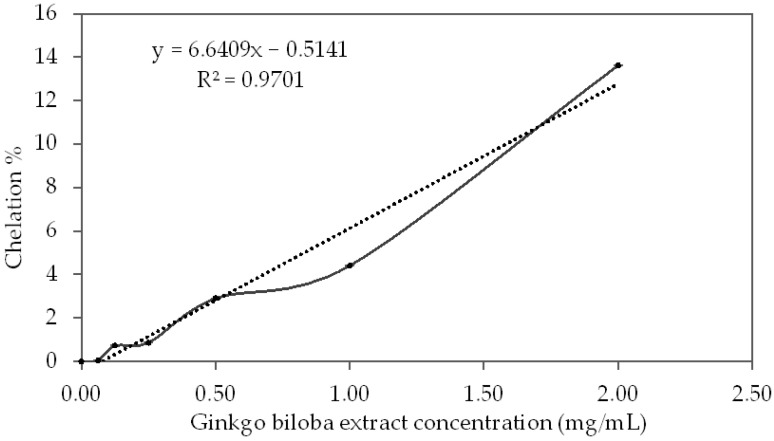
The curve of the chelation activity of iron ions from different concentrations of the *Ginkgo biloba* seed extract.

The results for the metal-chelating properties of GBSE are presented in Figure 6.

The degree of chelation of iron ions from different concentrations of GBSE is well described (R^2^ = 0.9701) with the following linear equation:

Chelation % = 6.6409x − 0.5141; where x is GBSE concentration (mg/mL)

The obtained calibration line for metal-chelating capacity with different concentrations of EDTA is described (R^2^ = 0.9966) with the following linear correlation equation:

Metal-chelating capacity (%) = 457.4x − 2.5082; x is the EDTA concentration (mM)

With a concentration of GBSE = 1.0 mg/mL, the calculated metal-chelating capacity was equal to 6.13%, and by substituting the above equation with this value, we could calculate that the chelating effect of 1 mg/mL GBSE was equal to EDTA at a concentration 0.0189 mM, from which we can calculate:

1 g/L GBSE = 0.0189 mM EDTA

(mmol EDTA)/(g GBSE) = 0.0189

## 4. Discussion

In this study, we investigated the phytochemical profile and the redox modulating potential—radical-scavenging, metal-reducing, and metal-chelating properties of methanol extract from seeds of *G. biloba* growing in Bulgaria’s Plovdiv region. The existing data have shown significant differences in the measured antioxidant activity of the *G. biloba* extracts depending on the used raw material, method of extraction, solvent, and region of growth even in the same country [30,31]. The antioxidant capacity of plant extracts is most often associated with the presence of secondary metabolites of the polyphenol group. Evidence of this is the good correlation found between the content of phenolic compounds and the antioxidant properties of extracts of different plant species, evaluated by applying various standardized antioxidant methods [32]; therefore, studies on the chromatography profile of the extracts and identification of the metabolites that could determine the antioxidant properties are needed in order to obtain standardized preparations with certain qualities.

The data of the chromatographic analysis of GBSE in this study showed the presence of two types of polyphenol representatives—flavonoids and terpenes. The established phytochemical composition of the methanol extract of seeds is similar to that of leaf extracts but differs in proportions [33]. In accordance with the literature data, terpenes (the triterpene ginkgolides A, B, C, and J, and the sesquiterpene bilobalide) prevail in the seed extract, while glycosylated flavonoids predominate in *G. biloba* leaf extracts [4]. In the standardized extract EGb 761^®^ (Tanakan^®^), commercially available in European markets, flavonoids constitute 24% and terpenes constitute 6% [34]. It has been established that terpenes isolated from *G. biloba* leaf have a well-defined antioxidant activity with possibilities for therapeutic applications in pathologies with oxidative stress etiology [13], although it has been shown that ginkgolides have less ability to scavenge DPPH radicals than ginkgo flavonoids [35]. The good therapeutic effect of terpenes in neurodegenerative diseases is probably due to the activation of various signaling pathways to protect cells from oxidative stress injury, in addition to their direct antioxidant action [13,36]. Flavonoids are the major components identified in *Ginkgo biloba* leaf extracts (ranging from 23–35%) in comparison to terpenoids (ranging from 0.17–11.3% for bilobalide, ginkgolide A, ginkgolide B, and ginkgolide C) [2]. Of the flavonoids in this study, we detected the presence of rutin, quercetin, and isorhamnetin. The most abundant was rutin (quercetin-3-rhamnosyl glucoside) (Table 2). Assays for establishing the in vitro antioxidant activity of rutin have found that its DPPH radical scavenging activity is much stronger than that of butylated hydroxytoluene and is comparable to vitamin C, as well as it effectively inhibits lipid peroxidation [37]. Isorhamnetin and quercetin are among the most important ingredients in the *G. biloba* leaves, and are responsible for their therapeutic benefits [38,39]. Both have shown excellent antioxidant activity [40]. With an equivalent concentration, the activity of quercetin in scavenging either DPPH or ABTS radicals was stronger than the activity of isorhamnetin [41]. These stronger effects of quercetin are likely due to the presence of the phenolic hydroxyl group linked to the 3‘Carbon atom in the molecule, while the 3‘Carbon atom of isorhamnetin is linked with the methoxy group, and this is the only difference between them; however, isorhamnetin has demonstrated very similar to quercetin activity for inhibiting lipid peroxidation [41]. The phytochemical analysis of the tested GBSE herein indicated the presence of two toxic compounds—ginkgotoxin and ginkgolic acid (Table 2). Although data on content ginkgotoxin (4’-O-methylpyrodoxin) in different parts of Ginkgo tree are contradictory, it is considered that in seeds its amount is larger than in the leaves. Ginkgotoxin is also known as antivitamin B_6_ and in certain quantities can cause stomach pain, nausea, irritability, epileptic convulsions, and death in very rare cases [42]. The European Pharmacopoeia has not yet standardized the safe levels of ginkgoxin in extracts. Concerning another toxic constituent of GBSE, ginkgolic acid, allergenic and genotoxic effects have been shown [43]. Initial analysis for ginkgolic acids in the GBSE tested in this study showed a concentration of 18 µg/g, which significantly exceeded the permissible levels set by the European Pharmacopoeia for leaf extract; however, ginkgolic acid isomers could be reduced without significant changes in the flavonoids and terpenes levels, as herein was performed.

The antioxidant activity of polyphenols involves different mechanisms: hydrogen atom transfer, single electron transfer, sequential proton loss electron transfer, and chelation of transition metals [44]. In this study, the widely accepted methods based on single-electron transfer and/or hydrogen atom transfer, such as ABTS, DPPH, FRAP, CUPRAC, as well as metal chelation tests, were applied. The obtained results demonstrated excellent antioxidant activity of the GBSE compared to leaf extracts when ABTS and FRAP methods were applied. The ABTS radical scavenging effect of the aqueous extract of Ginkgo biloba leaves obtained by the “reflux” method showed that at a final extract concentration of 100 µg/mL, 47.96 ± 0.40% of the ABTS radicals are neutralized [45]. This final concentration corresponds to the resulting concentration in our experiment since the sample with an initial concentration of 2.00 mg/mL was diluted 20-fold and became 100 µg/mL. At this concentration in our experiment, we found almost twice as much neutralization of ABTS radicals—95.6% ± 0.5%. Some data for the iron-reducing capacity of the leaf extracts of *Ginkgo biloba* obtained by the FRAP method showed that at a final extract concentration of 50 µg/mL, it was equivalent to 74.70 ± 1.71 µM FeSO_4_ [45]. At the same concentration (the initial concentration of 1.00 mg/mL GSBE was diluted 20-fold in the sample), we found that iron-reducing capacity was ten times greater, equivalent to only 7.33 µM FeSO_4_.

The antioxidant properties of the *Ginkgo biloba* leaf extracts studied by the DPPH method vary depending on the extraction procedure, growing region, and methods for calculation of the results. There are data demonstrating that leaf extract at a final concentration of 100 µg/mL and of 1000 µg/mL neutralized 58.47 ± 1.17% and 89.84 ± 2.11% of the DPPH radicals, respectively [45]. The above-mentioned concentrations correspond to the concentrations in our experiment (initial concentrations of 0.25 and 2.00 mg/mL with a two-fold dilution in the sample), at which we obtained 14.0 ± 0.88% and 80.1 ± 0.63% DPPH radical inhibition. The observed differences in the antioxidant effect at concentrations of 100 µg/mL in our experiment, compared to that at a similar concentration in the cited experiment, can be explained by the differences in the composition of the two extracts. Other authors [46] investigating the antioxidant properties of the methanol extracts of *Ginkgo biloba* leaves by the DPPH method found that at a final concentration of 150 µg/mL, 75.86% of DPPH radicals were neutralized. Actually, at a 150 µg/mL concentration, our extract would show only 7.8% neutralization of DPPH radicals. Ahmad et al. [47], in a comparative study of the antioxidant properties of methanolic and ethanolic extracts of *Ginkgo biloba* leaves by the DPPH method, found better antioxidant properties in methanol extract—82.8% compared to 76.7% for the ethanol extract, at the same concentration of 0.25 mg/mL. The methanol GBSE studied by us showed a close effect (80% inhibition) at four times higher concentration—2.0 mg/mL. When studying the antioxidant properties of leaf extracts of *Ginkgo biloba* from six regions of India by the DPPH method, the authors [30] obtained the lowest IC_50_ values for the extracts obtained by the “reflux” method (0.325 mg/mL) and the highest IC_50_ for the extracts obtained by the “shaker” method (6.5 mg/mL). The results for the extract obtained by the “reflux” method are comparable to the results for GBSE in our study—0.746 mg/mL. Values in the same range (max 1.545 ± 0.112 mg and min 0.394 ± 0.011 TEAC/g fresh matter) were measured for ethanol extracts of leaves from *Ginkgo biloba* growing in nine regions of Slovakia. The antioxidant activity assessed by the DPPH method could vary between extracts of the plant from different regions since the composition and biological activity of the compounds strongly depend on the agro-ecological conditions [31]. The significant difficulty in comparing the published results is the lack of a standard for determining the TEAC. TEAC is calculated by different methods, which cannot always be converted into each other.

The presence of free redox ions in biological systems, mainly Fe (II) and Cu (I), is also a possible source of reactive oxygen species generation and oxidative stress through the so-called Fenton reaction [23]; therefore, the use of compounds that may chelate these ions is also one of the antioxidant strategies. El-Beltagi et al. [46] investigated the metal-chelating effect of a methanolic extract of *Ginkgo biloba* leaves and found that at a concentration of 0.2 mg/mL, the extract chelated 32.2% of divalent iron ions. In our experiment, GBSE at a concentration of 0.25 mg/mL chelated only 0.9% of iron ions; however, it should be noted that the values given by the authors for the chelation of iron ions from the standard EDTA chelator differ significantly from those obtained by us. Thus, the authors find that at a concentration of 0.2 mg/mL (0.68 mM) EDTA chelates 51.2% of iron ions, and we obtain 99.3% chelation of iron ions at 0.5 mM EDTA [28]. In our opinion, it is necessary to establish standard methods for laboratory measurement and calculation of the antioxidant and other pharmacological properties of plant extracts for proper comparison and outlining of the expected therapeutic results. We could not compare the measured copper-reducing effect of GBSE because we did not find similar data in the literature.

These findings indicate that, along with the typical healing benefits of *Ginkgo biloba* seeds outlined by traditional Chinese medicine (pulmonary disorders, bladder inflammation, and enuresis), GBSE has antioxidant properties as a free radical scavenger and may be active in preventing free-radical-driven pathological reactions.

## 5. Conclusions

Recently, we have witnessed a resurgence of global interest in herbal medicines. More and more researchers are turning to the study of herbal products in healthcare; however, the concentrations of plant bioactive substances vary depending on a number of factors. In this study, we examined *Ginkgo biloba* seed extract, which is less used in practice but has the same biologically active substances as identified in the leaves. Our research focuses on the possibility of using this product in pathological conditions related to oxidative stress. In this study, we applied a panel of methods to investigate the redox-modulating activities of *Ginkgo biloba* seed extract containing complex organic compounds, including some with antioxidant potential.

The studied methanolic extract of *Ginkgo biloba* seeds was rich in flavonoids such as rutin and isorhamnetin and terpenes such as ginkgolides A, B, C, J, and bilobalide. Terpenes predominated in seed extract compared to flavonoids. Two toxic compounds have been identified in the crude extract—ginkgotoxin and ginkgolic acid. Their concentration can be reduced without reducing flavonoid and terpene levels. The studied Ginkgo biloba seed extract showed excellent properties for neutralizing ABTS and DPPH radicals, and the values found were comparable to those obtained by other authors with extracts of Ginkgo biloba leaves and nuts. The effects of iron reduction and iron chelation were significantly weaker than the published results for leaf extracts; however, in our opinion, these results are a promising start. Further studies of seed extracts in in vivo models of various pathological conditions are needed to fully determine their bioactivity and properties in order to understand the full phenomena of their healing potential.

## Figures and Tables

**Table 1:** Analytical conditions for detection of GBSE composition.

Analyte	Column	Mobile Phase	Gradient	Flow Rate [mL/min]	HESI-Ionizator Mode	Collision Energy [eV]	Scan Mode
**Flavonoids**	Syncronis C18 (150 × 4.6 mm, 5 µm); Thermo Fisher Scientific Inc., Waltham, MA, USA	А–0.1% HCOOH in МeCN–H_2_O (90:10, *v*/*v*) B–0.1% HCOOH in МeCN–H_2_O (10:90, *v*/*v*)	0–3 min–70% B, 3–10 min–42.5% B, 10–11 min–17.5% B, 11–19 min–0% B, 19–25 min–80% B	0.7	Negative	32 24 27 31	SRM
**Terpen trilactones**	Syncronis C18 (150 × 4.6 mm, 5 µm); Thermo Fisher Scientific Inc., Waltham, MA, USA	0.1% HCOOH in MeCN-H_2_O (50:50, *v*/*v*)	**Isocratic**	0.5	Negative	14 16 17 18 14	SRM
**Ginkgolic Acid** **C13:0**	Syncronis C18 (150 × 4.6 mm, 5 µm); Thermo Fisher Scientific Inc., Waltham, MA, USA	A-100% MeCN + 0.1% HCOOH B-10% MeCN + 0.1% HCOOH	0 min–30% B, 0–5 min–0 % B, 5–14 min–0% B, 14–25 min–30% B	1.0	Negative	23	SRM
**Ginkgotoxin**	Hypersil C18 (150 × 3 mm, 3 µm); Thermo Hypersil-Keystone Corp., Waltham, MA, USA	А-0.1% HCOOH in MeCN-H_2_O (60:40, *v*/*v*) B–0.1% HCOOH in МeCN–H_2_O (10:90, *v*/*v*)	**Reverse gradient** 0–1 min–80% B, 1–6 min–20% B, 6–15 min–10% B, 15–20 min–80% B	0.5	Positive	13	SRM

**Table 2:** Concentration (μg per gram of dry matter) and analytical characteristics of analyzed bioactive compounds in GBSE.

	№	Compound Name	Formula	RT [min]	Molecular Mass	Q1 ⟶ Q3 (*m*/*z*)	Detected Amount [µg/g]
**Flavonoids**	1.	Rutin	C_27_H_30_O_16_	3.36	610.50	609.2 ⟶ 300.5	27.59
	2.	Quercetin	C_15_H_10_O_7_	6.87	302.04	300.9 ⟶ 151.1	0.12
	3.	Kaempferol	C_15_H_10_O_6_	8.02	286.05	285 ⟶ 185.1	ND *
	4.	Isorhamnetin	C_16_H_12_O_7_	8.23	316.06	315 ⟶ 151	1.26
**Terpenes**	5.	Ginkgolide A	C_20_H_24_O_9_	5.67	408.14	453 ⟶ 407	104.67
	6.	Ginkgolides B, J	C_20_H_24_O_10_	5.66	424.14	423 ⟶ 367.5	221.44
	7.	Ginkgolide C	C_20_H_24_O_11_	3.79	440.13	439 ⟶ 383.02	47.52
	8.	Bilobalide	C_15_H_18_O_8_	5.25	326.10	324.9 ⟶ 163.0	103.69
	9.	Ginkgolic Acid C13:0	C_22_H_34_O_3_	12.08	346.50	319.2 ⟶ 275.3	3.90
	10.	Ginkgotoxin	C_9_H_13_NO_3_	7.11	183.20	183.97 ⟶ 152.04	125.05

* ND—not detected; Ginkgolides B and J are isomers and cannot be separated under the chromatographic conditions used [4].

## Data Availability

The authors confirm that the data supporting the findings of this study are available within the article. Row data that support the findings of this study are available from the corresponding author upon reasonable request.

## Supplementary Materials

The following supporting information can be downloaded at: https://www.mdpi.com/article/10.3390/life12060878/s1, Figure S1. Chromatograms of flavonoids standards: Kaempferol (1A), Quercetin (2A), Isorhamnetin (3A), Rutin (4A), and of flavonoids in GBSE: Kaempferol (1B), Quercetin (2B), Isorhamnetin (3B), and Rutin (4B); Figure S2. Chromatograms of terpenes standards: Bilobalide (1A), Ginkgolides B and J (2A), Ginkgolide C (3A), Ginkgolide A (4A), and of terpenes in GBSE: Bilobalide (1B), Ginkgolides B and J (2B), Ginkgolide C (3B), and Ginkgolide A (4B); Figure S3. Chromatogram of Ginkgolic Acid standard (A) and of Ginkgolic Acid in GBSE (B); Figure S4. Chromatogram of Ginkgotoxin standard (A) and of Ginkgotoxin in GBSE (B).
