# Supplementary material for: Chromatographic Profile and Redox-Modulating Capacity of Methanol Extract from Seeds of Ginkgo biloba L. Originating from Plovdiv Region in Bulgaria
Source: Life (Basel). 2022 Jun 12;12(6):878. doi: 10.3390/life12060878 (PMC9228083; doi:10.3390/life12060878)
Supplement: Supplementary file 1 [file life-12-00878-s001.zip › life-1749448-supplementary.pdf]

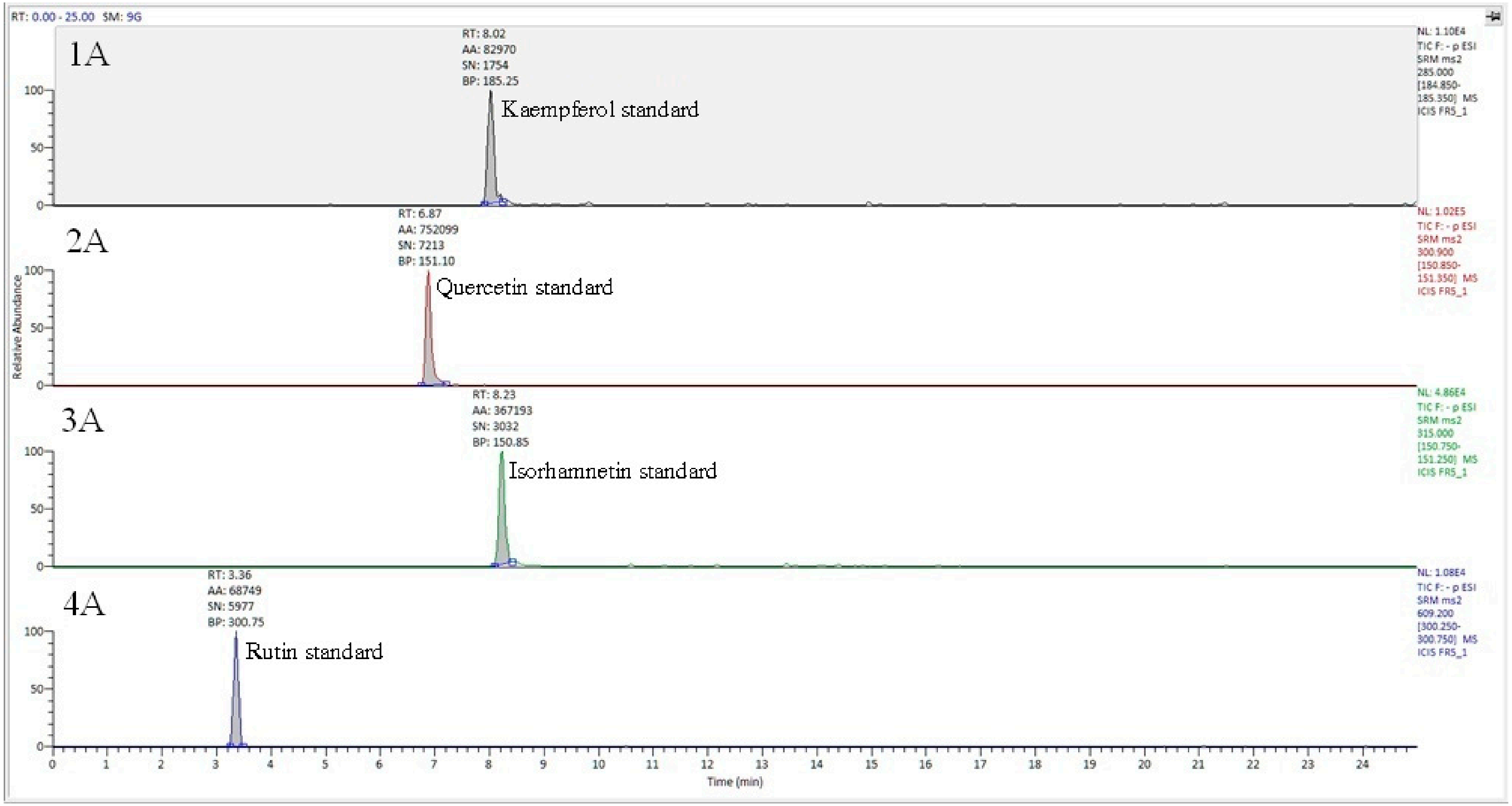

RT: 0.00 - 25.00 SM: 9G
1A
RT: 8.02
AA: 82970
SN: 1754
BP: 185.25
Kaempferol standard
NL: 1.10E4
TIC F: - p ESI
SRM ms2
285.000
[184.850-
185.350] MS
ICIS FR5_1
2A
RT: 6.87
AA: 752099
SN: 7213
BP: 151.10
Quercetin standard
NL: 1.02E5
TIC F: - p ESI
SRM ms2
300.900
[150.850-
151.350] MS
ICIS FR5_1
3A
RT: 8.23
AA: 367193
SN: 3032
BP: 150.85
Isorhamnetin standard
NL: 4.86E4
TIC F: - p ESI
SRM ms2
315.000
[150.750-
151.250] MS
ICIS FR5_1
4A
RT: 3.36
AA: 68749
SN: 5977
BP: 300.75
Rutin standard
NL: 1.08E4
TIC F: - p ESI
SRM ms2
609.200
[300.250-
300.750] MS
ICIS FR5_1
Relative Abundance
Time (min)

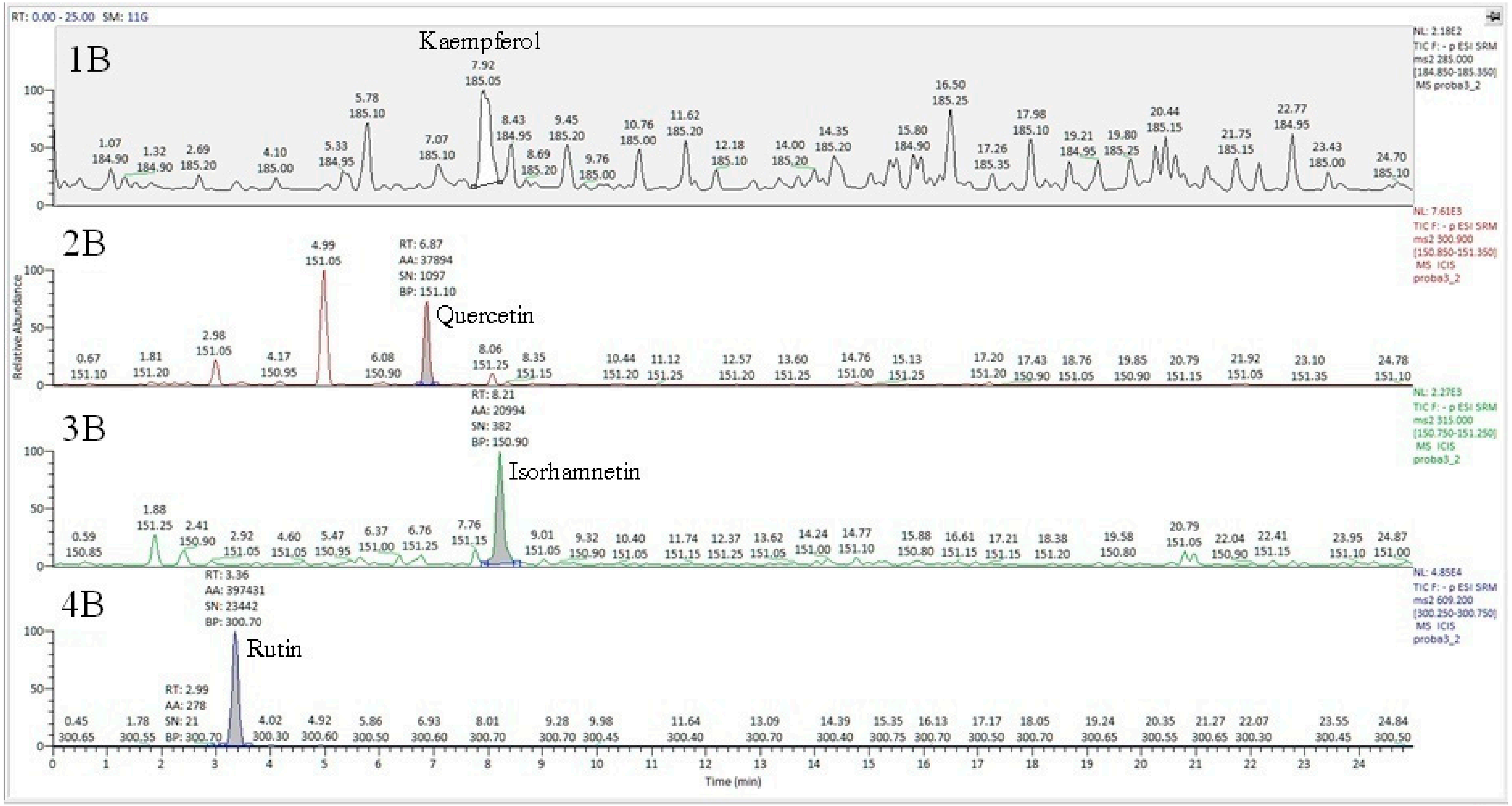

Figure S1. Chromatograms of flavonoids standards: Kaempferol (1A), Quercetin (2A), Isorhamnetin (3A), Rutin (4A) and of flavonoids in GBSE: Kaempferol (1B), Quercetin (2B), Isorhamnetin (3B), Rutin (4B).

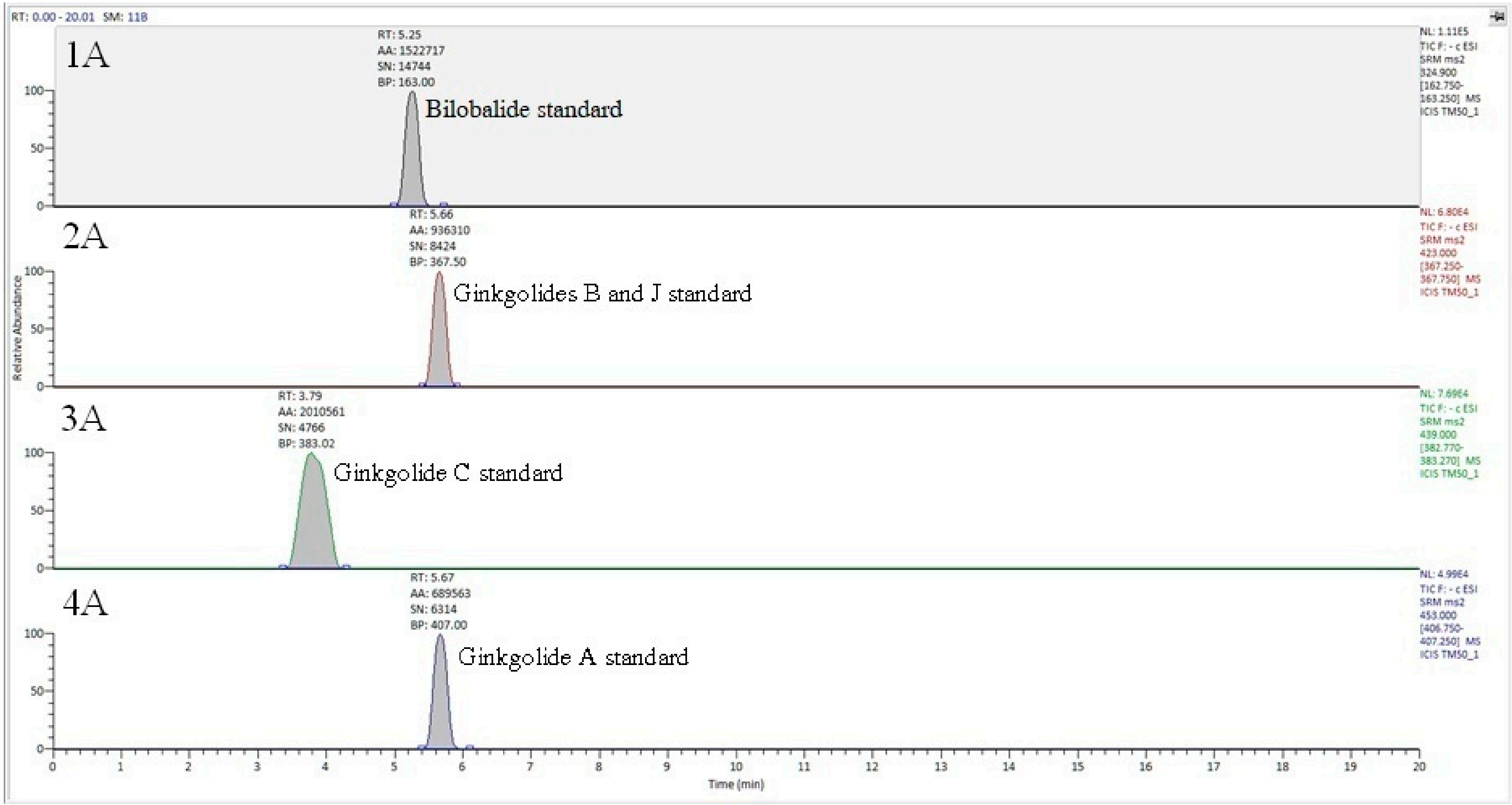

RT: 0.00 - 20.01 SM: 11B
1A
RT: 5.25
AA: 1522717
SN: 14744
BP: 163.00
Bilobalide standard
NL: 1.11E5
TIC F: - c ESI
SRM ms2
324.900
[162.750-
163.250] MS
ICIS TM50_1
2A
RT: 5.66
AA: 936310
SN: 8424
BP: 367.50
Ginkgolides B and J standard
NL: 6.80E4
TIC F: - c ESI
SRM ms2
423.000
[367.250-
367.750] MS
ICIS TM50_1
3A
RT: 3.79
AA: 2010561
SN: 4766
BP: 383.02
Ginkgolide C standard
NL: 7.69E4
TIC F: - c ESI
SRM ms2
439.000
[382.770-
383.270] MS
ICIS TM50_1
4A
RT: 5.67
AA: 689563
SN: 6314
BP: 407.00
Ginkgolide A standard
NL: 4.99E4
TIC F: - c ESI
SRM ms2
453.000
[406.750-
407.250] MS
ICIS TM50_1
Relative Abundance
Time (min)

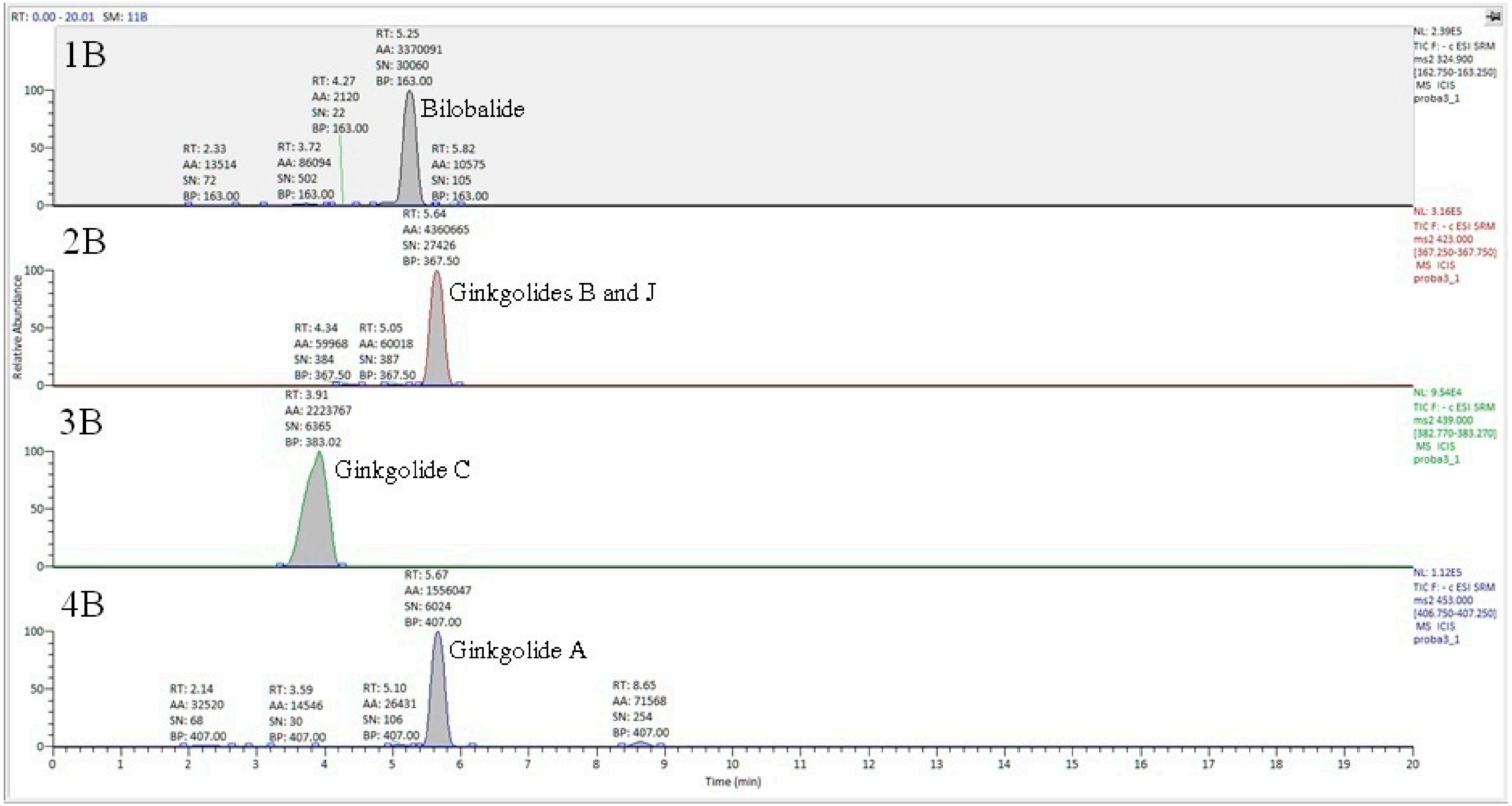

Figure S2. Chromatograms of terpenes standards: Bilobalide (1A), Ginkgolides B and J (2A), Ginkgolide C (3A), Ginkgolide A (4A) and of terpenes in GBSE: Bilobalide (1B), Ginkgolides B and J (2B), Ginkgolide C (3B), Ginkgolide A (4B).

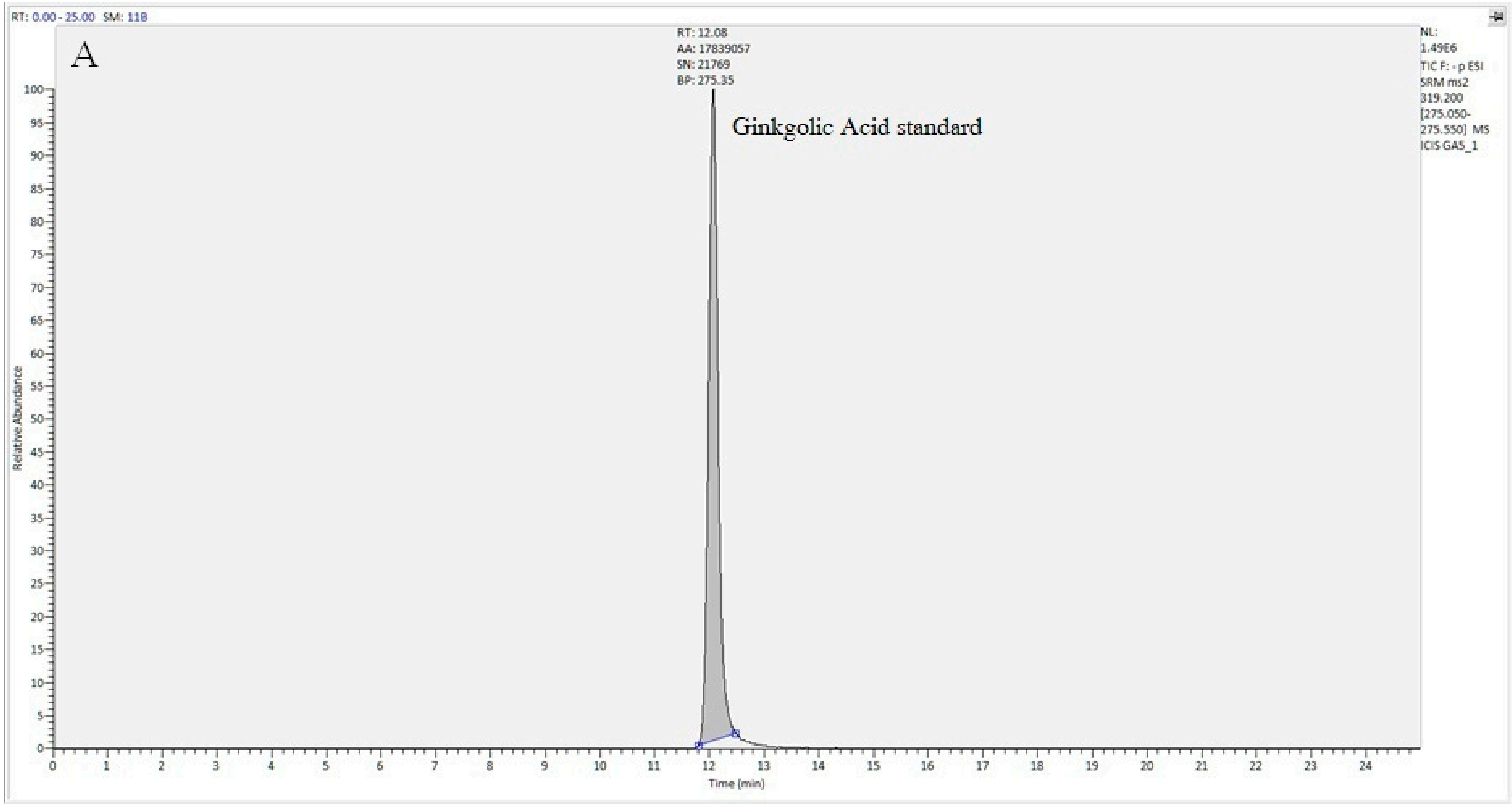
RT: 0.00 - 25.00 SM: 11B
A
RT: 12.08
AA: 17839057
SN: 21769
BP: 275.35
Ginkgolic Acid standard
NL:
1.49E6
TIC F: - p ESI
SRM ms2
319.200
[275.050-
275.550] MS
ICIS GA5_1
Relative Abundance
Time (min)

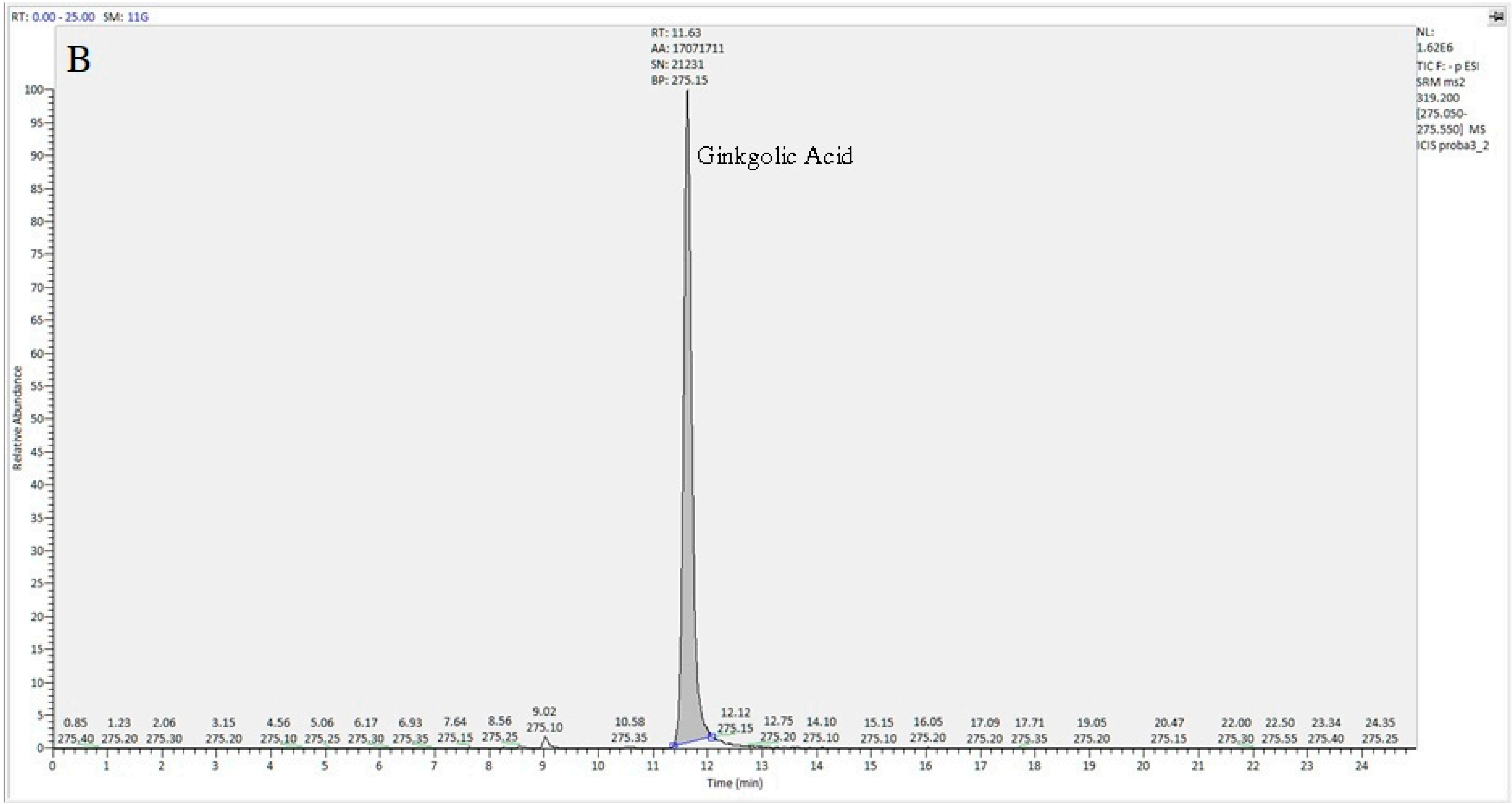

Figure S3. Chromatogram of Ginkgolic Acid standard (A) and of Ginkgolic Acid in GBSE (B).

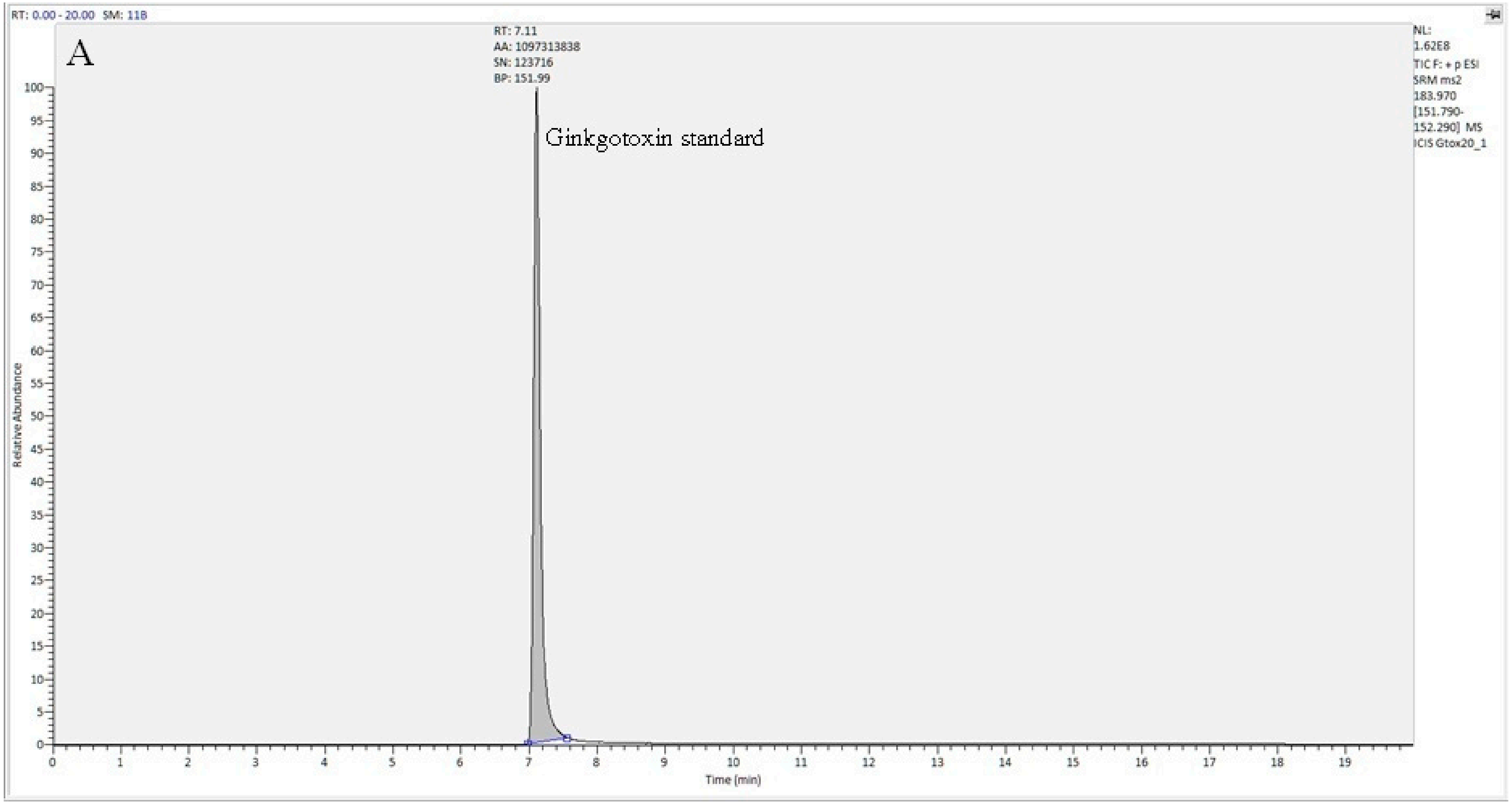
RT: 0.00 - 20.00 SM: 11B
A
RT: 7.11
AA: 1097313838
SN: 123716
BP: 151.99
Ginkgotoxin standard
NL:
1.62E8
TIC F: + p ESI
SRM ms2
183.970
[151.790-
152.290] MS
ICIS Gtox20_1
Relative Abundance
Time (min)

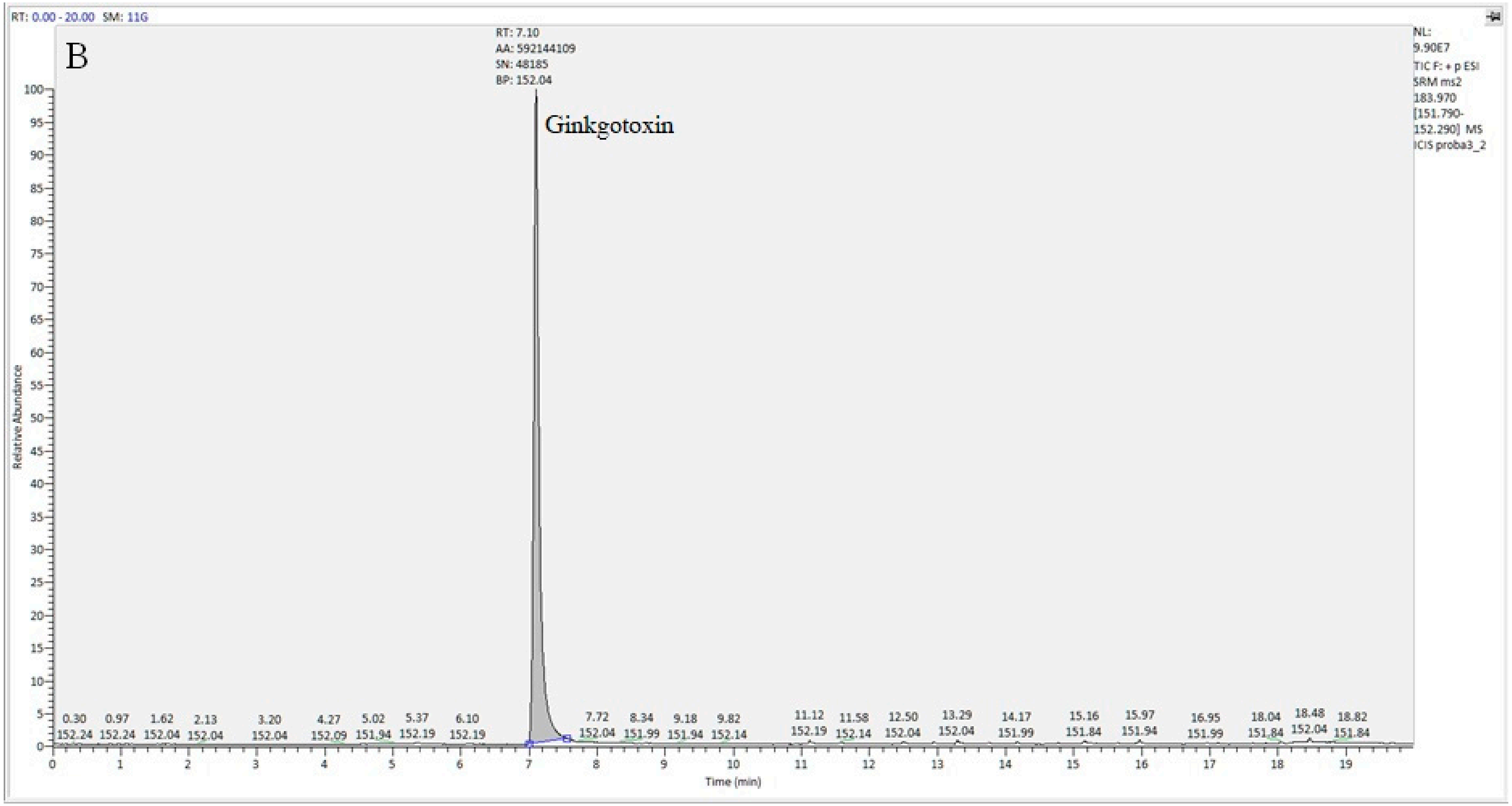

Figure S4. Chromatogram of Ginkgotoxin standard (A) and of Ginkgotoxin in GBSE (B).
